# A Novel Herbal Paste Formulation of Turmeric, Tulsi, and Honey for the Treatment of Oral Submucous Fibrosis

**DOI:** 10.7759/cureus.46608

**Published:** 2023-10-06

**Authors:** Shaik Mobeen, Ravindra SV, Sunitha JD, Rathod Prakash, Satyanarayana D, Himaja Swayampakula, Afeefa Shaikh, Amreen Begum

**Affiliations:** 1 Oral Medicine and Radiology, MNR Dental College and Hospital, Sangareddy, IND; 2 Oral and Maxillofacial Pathology, MNR Dental College and Hospital, Sangareddy, IND; 3 Oral and Maxillofacial Surgery, MNR Dental College and Hospital, Sangareddy, IND; 4 Public Health Dentistry, MNR Dental College and Hospital, Sangareddy, IND; 5 Prosthodontics, MNR Dental College and Hospital, Sangareddy, IND

**Keywords:** anti-oxidant property, restricted mouth opening, burning sensation, traditional ayurvedic medicine, oral sub mucous fibrosis

## Abstract

Background and objectives

Oral submucous fibrosis (OSMF) is a condition that affects the oral cavity and is characterized by the development of fibrous bands in the submucosal layers, leading to progressive difficulty in mouth opening and other symptoms. Inflammation and advancing fibrosis of the submucosal tissues are the hallmarks of this chronic, disabling illness of the oral cavity. The disorder is well known for having a propensity for cancer and is particularly linked to the habit of chewing tobacco and areca nuts.

The study mentioned aims to compare the effectiveness of a novel herbal paste formulation containing turmeric, tulsi (holy basil), and honey in managing OSMF. The use of herbal remedies is common in traditional medicine, and turmeric and tulsi are known for their potential anti-inflammatory and antioxidant properties. Honey, too, is believed to have some therapeutic benefits.

Methodology

A study was conducted at MNR Dental College and Hospital, Sangareddy, Telangana, India, in the Department of Oral Medicine and Radiology (OMR) on 80 oral submucous fibrosis patients to evaluate the efficacy of a novel herbal paste formulation of turmeric, tulsi, and honey, comprising 77 males and three females. Patients were given a novel herbal paste formulation consisting of 10 g of turmeric powder and 10 g of tulsi powder, mixed in 10 ml of honey, to study subjects, and a placebo consisting of anti-oxidants was administered to control subjects for three months. The subjective and objective symptoms were recorded and statistically analyzed.

Results

The majority of the subjects were in the age group of 18-22 years, with an average age of 28.09+/-8.38 years with the most predominant habit of gutka chewing.

A statistically significant change in the mouth opening, tongue protrusion, burning sensation, blanching of the mucous membrane, and reduction in palpable fibrous bands was found in the study subjects when compared to control subjects.

Conclusion

The present study evaluated the efficacy of a novel herbal paste formulation of turmeric, tulsi, and honey in the management of OSMF.

## Introduction

Oral submucous fibrosis (OSMF) is a condition that affects the oral cavity and is characterized by the development of fibrous bands in the submucosal layers [1]. This leads to progressive difficulty opening the mouth, in addition to other symptoms. This condition is well-known for its malignant potential, meaning it can lead to oral cancer if not managed properly.

The main risk factors associated with OSMF are the chewing of areca nuts and tobacco. These habits are particularly prevalent in Southeast Asia and India, where they have existed for thousands of years. Areca nut is commonly chewed with betel leaf and other ingredients, and this mixture is known by various names depending on the region, such as "pan" or "paan" in India.

Continuous chewing and contact with these substances can lead to chronic irritation and inflammation of the oral tissues, which eventually lead to the formation of fibrous bands and a restricted opening of the mouth. This condition can significantly impair a person's ability to eat, speak, and perform other oral functions.

Pindborg further emphasizes the chronic and progressive nature of this condition, which affects various parts of the oral cavity and, at times, even the pharyngeal mucosa [2].

The initial symptoms of OSMF, such as a burning sensation in the oral cavity and nonspecific vesicular stomatitis, can be subtle and easily overlooked, leading to a delayed diagnosis. As the disease progresses, the characteristic fibrosis of the cheeks, lips, tongue, or palate becomes apparent, leading to the formation of vertical bands running through the mucosa. The fibrotic changes cause stiffness and loss of elasticity in the oral mucosa, resulting in trismus, or difficulty opening the mouth, which can severely impact a person's ability to eat and speak.

The severity of the fibrosis can progress to the extent that the affected areas appear white and become firm, leading to significant restrictions in opening the mouth and limited mobility of the tongue and/or palate. This can have a considerable impact on a person's quality of life.

Moreover, the association of OSMF with epithelial atrophy and its malignant potential must not be underestimated [3]. It has been observed that OSMF may transform into oral cancer, and the risk is reported to be as high as 8%. This underscores the importance of early detection, regular monitoring, and appropriate management of this condition to prevent its progression to oral cancer [4].

Oral submucous fibrosis is a serious health concern, and its association with areca nut and tobacco chewing highlights the importance of addressing these harmful habits to reduce the risk of developing this condition and oral cancer. Timely intervention, lifestyle modifications, and cessation support can make a significant difference in the management and prevention of OSMF-related complications.

Therefore, the present study was conducted to determine the efficacy of a novel herbal paste formulation of turmeric, tulsi (holy basil), and honey as a conservative means in the management of OSMF.

## Materials and methods

The present study was conducted in the Department of Oral Medicine and Radiology (OMR), MNR Dental College and Hospital, Sangareddy, Telangana, India, to evaluate the efficacy of a novel herbal paste formulation of turmeric, tulsi, and honey in the management of OSMF. Institutional Ethical Committee approval was obtained from the MNR Institutional Ethical Committee before starting the study (ref no.: MNR/DC-IEC/001/2022). Informed consent was obtained from each patient for participation in this study.

Aims of the study

An open-label clinical study was conducted to assess the safety and effectiveness of a novel herbal paste formulation of turmeric, tulsi, and honey in the management of OSMF. This herbal paste contains 10 mg of turmeric powder (*Curcuma longa*), 10 mg of tulsi powder, and 10 ml of honey. The study aims to evaluate its potential for treating OSMF.

Inclusion criteria

The inclusion criteria were patients with clinically and histopathologically proven OSMF, involving the following characteristics: a positive history of chewing areca nut or one of its commercial preparations; difficulty in swallowing and chewing; a burning sensation while consuming spicy foods; mouth opening >/=30 mm; and changes observed in the oral mucous membrane-blanching, stiffness, and the presence of vertical fibrous bands.

Exclusion criteria

The exclusion criteria included the following aspects: mouth opening <30 mm; the presence of other systemic illnesses such as diabetes mellitus and hypertension in addition to other aspects; other mucosal lesions; and subjects who refused to undergo biopsy.

Materials

For the study, 80 subjects diagnosed clinically with OSMF were selected from the OPD of the department of OMR, MNR Dental College, and Hospital, Sangareddy. Among them, 40 subjects were randomly categorized as study subjects and the other 40 as control subjects. The participants, consisting of 37 males and three females, were then evaluated clinically for submucous fibrosis using a mouth mirror and probe. Before the study, each patient received a detailed explanation about the study's purpose, and written informed consent and institutional ethical committee clearance were obtained.

Methodology

The herbs were procured from the local markets in Hyderabad and identified by the botanists of the Central Council for Research in Unani Medicine (CCRUM), Hyderabad, by conducting a pharmacognosy study. They were powdered by a pulverizer machine in the MNR Foundation for Research and Innovations (MNR-FRI) Lab. The rhizomes of *Curcuma longa*, leaves of tulsi, and natural honey were used. The composition of the honey used was carbohydrates, comprising a major portion of 79%; fructose (48%), glucose (45%), sucrose (1%), others (6%), water (22%), enzymes, and free amino acids, all of which were abundant in proline, trace amounts of vitamin B, minerals, and antioxidants like flavonoids and vitamin C. The patients were advised to mix 10 grams of turmeric powder and 10 grams of tulsi powder in 10 ml of honey to make a paste. The patients were instructed to apply the paste to the oral mucosa four times per day. The instructions given to the patient were not to eat or drink anything for 10 minutes after the application of the paste. The 40 study subjects were given instructions about the study procedure, investigations, and follow-up visits every 15 days for a total of 90 days. On follow-up visits every 15 days during the 90-day duration of the study, the patients were asked to bring the used packets back to check their compliance. The patients were asked to report immediately if they experienced any adverse or unfavorable reaction or any discomfort after the application of the paste. The 40 control subjects received placebo treatment with a Plexavit (8 mg) soft gel capsule, once daily, for 90 days. Similar to the study subjects, the control subjects were also evaluated every 15 days for about 90 days for both subjective and objective symptoms.

Objective symptoms

The objective symptoms were improvements in mouth opening, tongue protrusion, blanching, vertical fibrous bands, and changes in the shape of the uvula.

Subjective symptoms

Relief from intolerance to spicy foods and relief from burning sensations were some of the subjective symptoms evaluated. Various other subjective symptoms were also considered. The measurement of the mouth opening was determined by the interincisal distance, which was measured from the mesio-incisal edge of the upper left central incisor tooth to the mesio-incisal edge of the lower left central incisor tooth. The distance was calculated using a divider and scale and recorded in millimeters (mm). The function of the tongue was evaluated for movement and protrusion. A positive recording was made when the patient reported restricted tongue protrusion, followed by visual observation of the protruded tongue from the lateral aspect of the head, which means viewing the tongue from the side. Additionally, the distance from the mesial contact area of the lower central incisors (the point where the edges of the lower front teeth meet) to the tip of the protruded tongue was measured. This measurement helped quantify the extent of tongue protrusion and provided valuable information about the patient's tongue mobility.

The involvement of the uvula was recorded as positive when it appeared shrunken or deviated, with or without blanching. The presence of fibrous bands was recorded as positive when areas of the right and left buccal mucosa, right and left vestibules, faucal pillars, soft palate, lips, and the floor of the mouth exhibited a lack of suppleness, the presence of palpable fibrous bands, or marked stiffness. The patient's response to the burning sensation was recorded as present, reduced, or absent at 15-day intervals. Blanching was visually assessed and recorded as present or reduced.

## Results

Table 1 presents the distribution of sex in both study subjects and control subjects.

**Table 1:** Distribution of sex in the study subjects and control subjects

Sex	Study subjects	%	Control subjects	%	Total	%
Male	37	92.50%	40	100.00	77	96.25
Female	3	7.50%	0	0.00	3	3.75
Total	40	100.00	40	100.00	80	100.00

The mean age of the study subjects was 29 years, while the mean age of the control subjects was 27 years. Among the participants, there were 37 males in the study subjects and 40 males in the control subjects. Table 2 shows the distribution of age in the study subjects and control subjects.

**Table 2:** Distribution of age in both study subjects and control subjects SD: standard deviation

Age group (in years)	Study subjects	%	Control subjects	%	Total	%
18–22	12	30.00	14	35.00	26	32.50
23–27	9	22.50	12	30.00	21	26.25
28–32	9	22.50	6	15.00	15	18.75
33+	10	25.00	8	20.00	18	22.50
Total	40	100.00	40	100.00	80	100
Mean age (in years)	29.15		27.03		28.09	
SD age (in years)	9.07		7.59		8.38	

Here, the age range of 18-22 years comprised 12 (30%) study subjects and 14 (35%) control subjects. A total of 26 (32.50%) subjects were in this range. The age range of 23-27 years comprised nine (22.50%) study subjects and 12 (30%) control subjects. A total of 21 (26.25%) subjects were in this range. The age range of 28-32 years comprised nine (22.50%) study subjects and six (15%) control subjects. A total of 15 (18.75%) subjects were in this group. The age group above 33 years comprised 10 (25%) study subjects and eight (20%) control subjects. A total of 18 (22.50%) subjects were present in this group. The mean age for the study subjects was 29.15+/−9.07 years, the mean age for the control subjects was 27.03+/−7.59 years, and the total mean age was 28.09+/−8.38 years.

Table 3 shows the distribution of the study subjects and the control subjects by habit.

**Table 3:** Distribution of the study and control subjects by habit These are commercial preparations of pan masala.

Type of habit	Study subjects	%	Control subjects	%	Total	%
Betel quid	4	10.00	7	17.50	11	13.75
Dilbag	17	42.50	13	32.50	30	37.50
Gagan	7	17.50	15	37.50	22	27.50
Kuber	12	30.00	5	12.50	17	21.25
Total	40	100.00	40	100.00	80	100.00

Among the study subjects, four (10%) used betel quid, 17 (42.5%) used Dilbag, seven (17.5%) used Gagan, and 12 (30%) used Kuber. Among the control subjects, seven (17.5%) used betel quid, 13 (32.5%) used Dilbag, 15 (37.5%) used Gagan, and five (12.5%) used Kuber. These are commercial preparations of pan masala.

Table 4 compares study and control subjects based on mouth-opening scores at the first visit and the last visit.

**Table 4:** Comparison of the study and the control subjects based on the mouth-opening scores at the first and last visits and their difference by t-test Statistically significant p-value<0.005

Variable	Group	Mean (in mm)	SD (in mm)	t-value	p-value
First visit	Study subjects	33.53	4.81	0.4088	0.6838
	Control subjects	33.18	2.48		
Last visit	Study subjects	35.93	4.97	3.1296	0.0025*
	Control subjects	33.18	2.48		
Difference	Study subjects	2.40	1.71		
	Control subjects	0.00	0.00	8.8937	0.0000*

**Figure 1:**
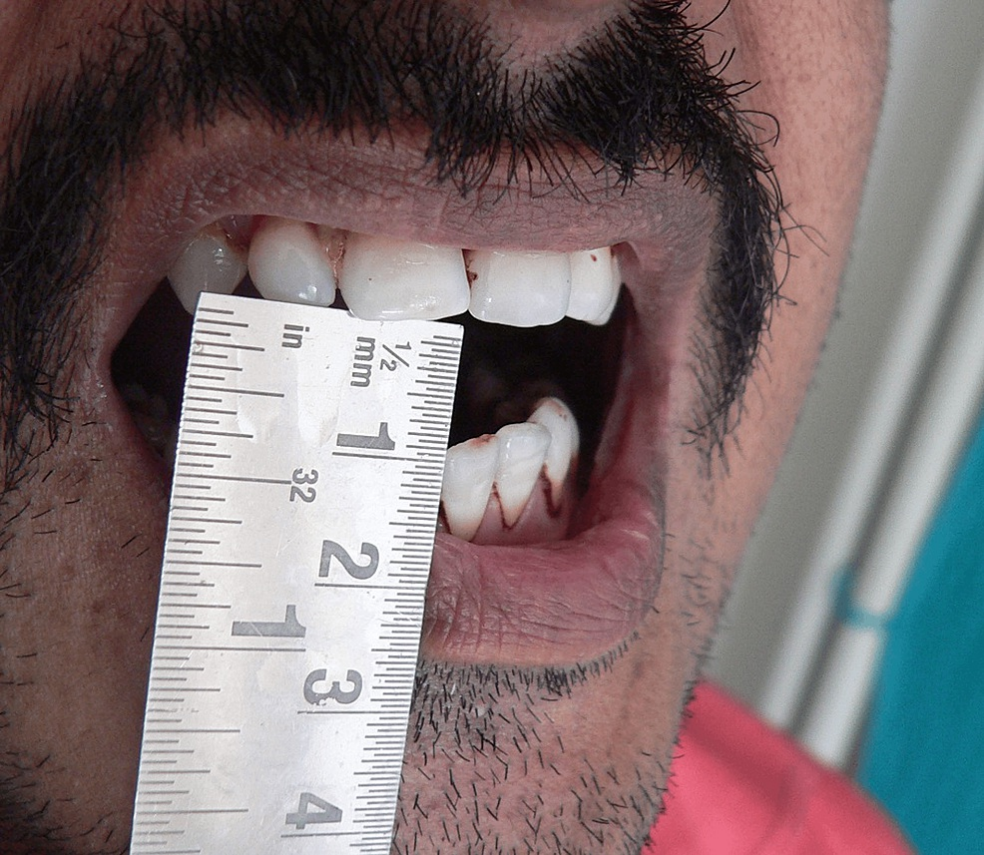
Pre-treatment mouth-opening picture

**Figure 2:**
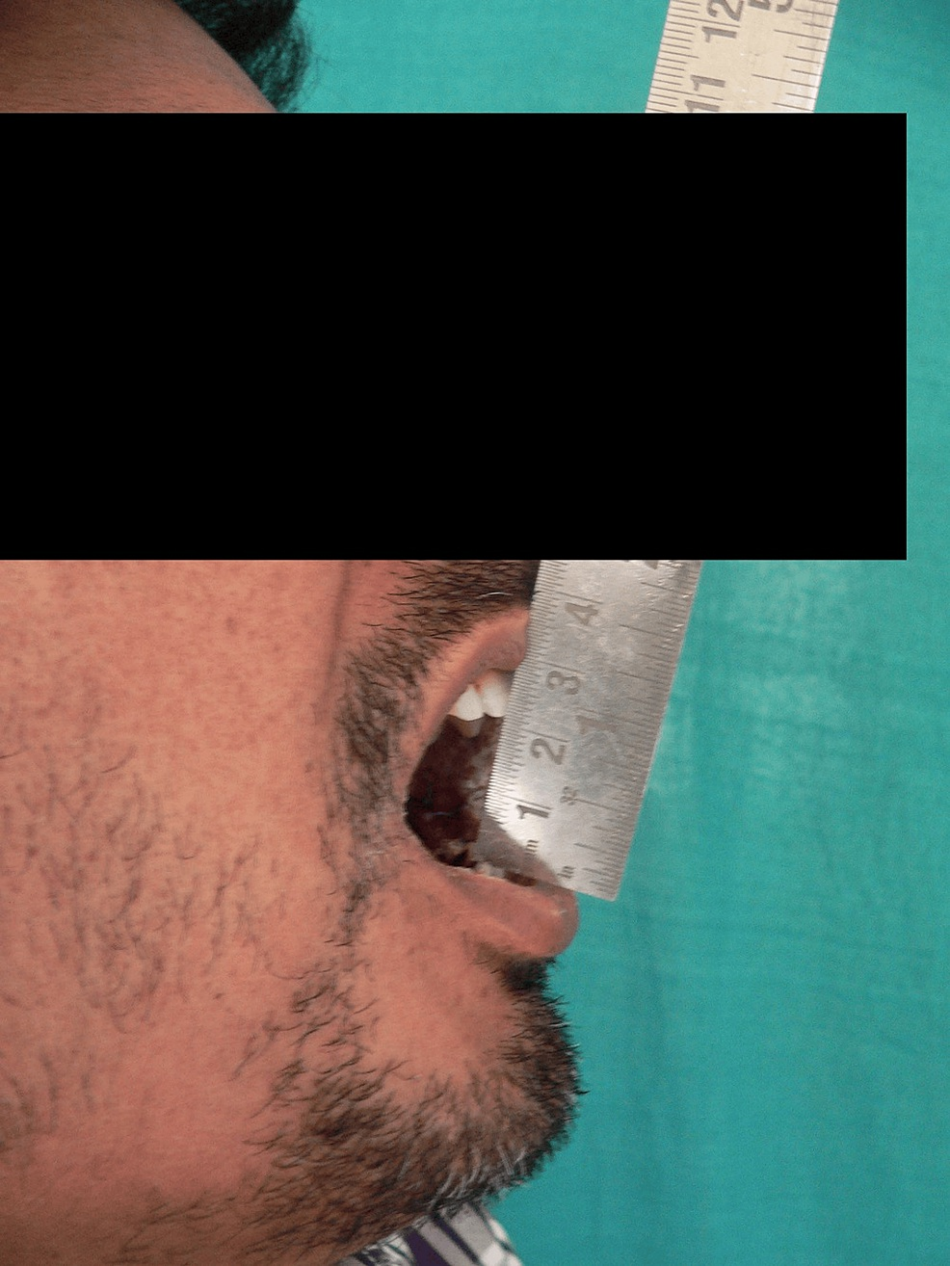
Picture of mid-treatment improvement in mouth opening

**Figure 3:**
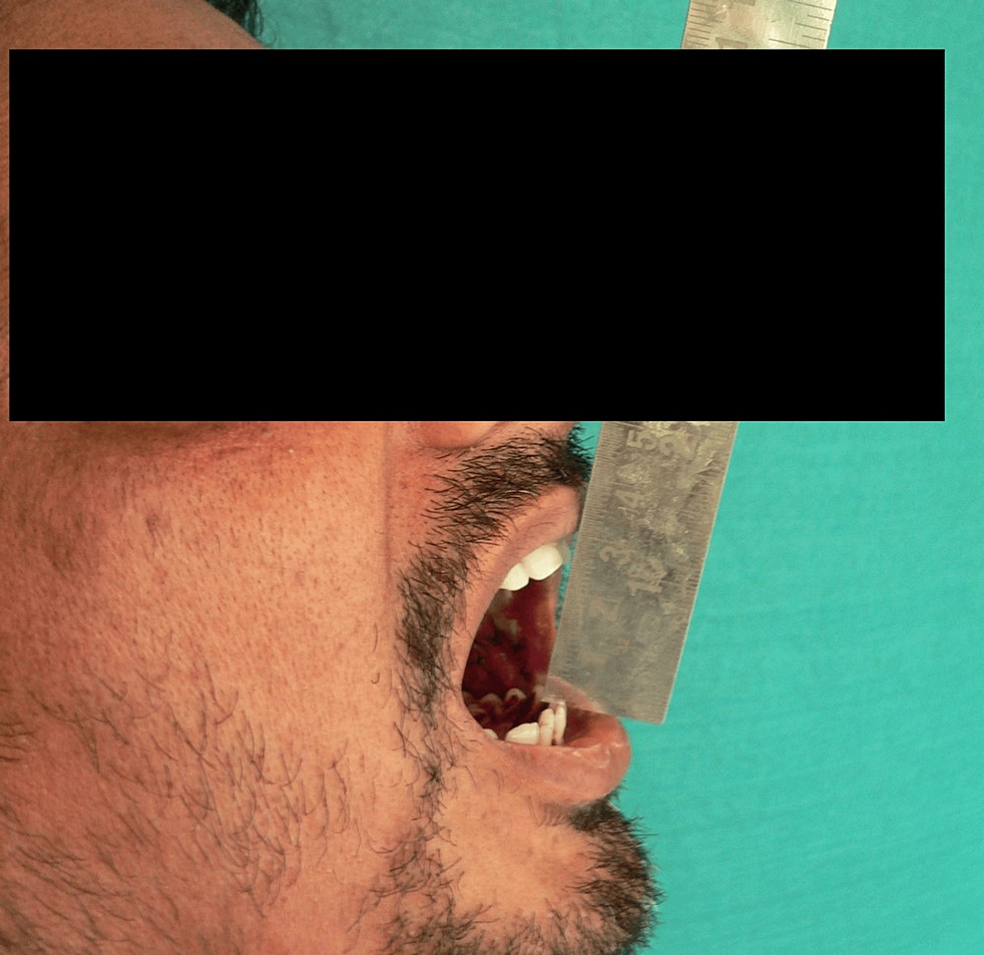
Picture of post-treatment improvement in mouth opening

On the first visit, the average mouth-opening score in the study subjects was 33.53 mm+/−4.81 mm, and the average mouth-opening in the control subjects was 33.18 mm+/−4.97 mm. The average mouth opening in the control subjects was 33.18 mm+/−2.48 mm. The statistical analysis showed a highly significant p-value <0.005 (Figures 1-3).

Table 5 compares the tongue-protrusion scores between the study and control subjects at the first visit and the last visit.

**Table 5:** Comparison of study and control subjects' tongue-protrusion scores at the first visit and the last visit and their difference by t-test Statistically significant p-value <0.005

Variable	Group	Mean (in mm)	SD (in mm)	t-value	p-value
First visit	Study subjects	48.23	8.38	−6.1973	0.000*
	Control subjects	57.13	3.50	-	
Last visit	Study subjects	49.33	7.93	−5.6903	0.000*
	Control subjects	57.13	3.50	-	
Difference	Study subjects	1.10	1.45	4.8096	0.000*
	Control subjects	0.00	0.00		

On the first visit, the average tongue protrusion in the study subjects was 48.23 mm+/−8.38 mm, and in the control subjects, it was 57.13 mm+/−3.50 mm. In the last visit, the average tongue protrusion in study subjects was 49.33 mm+/−7.93 mm, and in control subjects, it was 57.13 mm+/−3.50 mm. The statistical analysis was found to be significant with a p-value of 0.000.

Table 6 compares the first visit and the last visit with mouth-opening scores between the study and the control subjects by paired t-test.

**Table 6:** Comparison of the first visit and last with mouth-opening scores in study and control subjects by paired t-test *statistically significant p-value <0.005

Groups	Visit	Mean (in mm)	SD (in mm)	Mean diff (in mm)	SD diff (in mm)	% of change	Paired t-test	p-value
Study	First visit	33.53	4.81	−2.40	1.71	−7.16	−8.8941	0.000*
	Last visit	35.93	4.97	-	-	-	-	0.000*
Control	First visit	33.18	2.48	0.00	-	0.00	0.000	1.000
	Last visit	33.18	2.48	-	-	0.00	0.000	1.000

The average mouth-opening score for study subjects on the first visit was 3.53 mm+/−4.81 mm and on the last visit was 35.93 mm+/−4.97 mm and showed a mean difference of −2.40 mm+/−1.71 mm with −7.61% of change. The statistical analysis showed it to be highly significant with a paired t-test (−8.8941) and p-value (0.00000). The average mouth-opening score for the first visit in the control subjects was 33.18 mm+/−2.48 mm, and the same score for their last visit was 33.18 mm+/−2.48 mm. The mean difference was 0.00 mm with a 0.00% change.

Table 7 compares the first visit and last visit with tongue-protrusion scores between the study and control subjects by paired t-test.

**Table 7:** Comparison of the tongue protrusion scores in the first and last visits in the study and control subjects by paired t-test *statistically significant p-value <0.005

Group	Visit	Mean (in mm)	Std dev (in mm)	Mean diff (in mm)	SD diff (in mm)	% of change	Paired t-test	p-value
Study	First visit	48.23	8.38	−1.10	1.45	−2.28	−4.810	0.000*
	Last visit	49.3	7.93	-	-	-	-	0.000*
Control	First visit	57.13	3.50	0.00	-	0.00	0.0000	1.0000
	Last visit	57.13	3.50	-	-	0.00	0.0000	1.0000

In the first visit, the average tongue protrusion in the study subjects was 48.23 mm+/−8.38 mm, and in the last visit, the average tongue protrusion in the study subjects was 49.3 mm+/−7.93 mm. The mean difference was −1.10 mm+/−1.45 mm, with a −2.28% change. The statistical analysis showed it to be highly significant, with a paired t-test value of −4.8101 and a p-value of 0.000. In the first visit, the average tongue protrusion in the control subjects was 57.13 mm+/−3.50 mm, and in the last visit, the score was 57.13 mm+/−3.50 mm. The mean difference was 0.00 mm with a 0.00% change. The statistical analysis showed it to be insignificant with a p-value of 1.000.

Table 8 compares the study and control subjects with the status of blanching at the first and last visits.

**Table 8:** Comparison of the status of blanching experienced by study and control subjects at the first and last visits Between the present and reduced in the last visit: Chi-square with Yates’s correction = 4.5051, p = 0.0341*; Between the first visit and the last visit in the study subjects: Z = 2.2013; p = 0.0277*; Between the first visit and last visit in the control subjects: Z = 0.0000, p = 1.0000

Visit	Blanching	Study subjects	%	Control subjects	%	Total	%
First visit	Present	40	100.00	40	100.00	80	100.00
	Reduced	0	0.00	0	0.00	0	0.00
Last visit	Present	34	85.00	40	100.00	74	92.50
	Reduced	6	15.00	0	0.00	6	7.50
	Total	40	100.00	40	100.00	80	100.00

**Figure 4:**
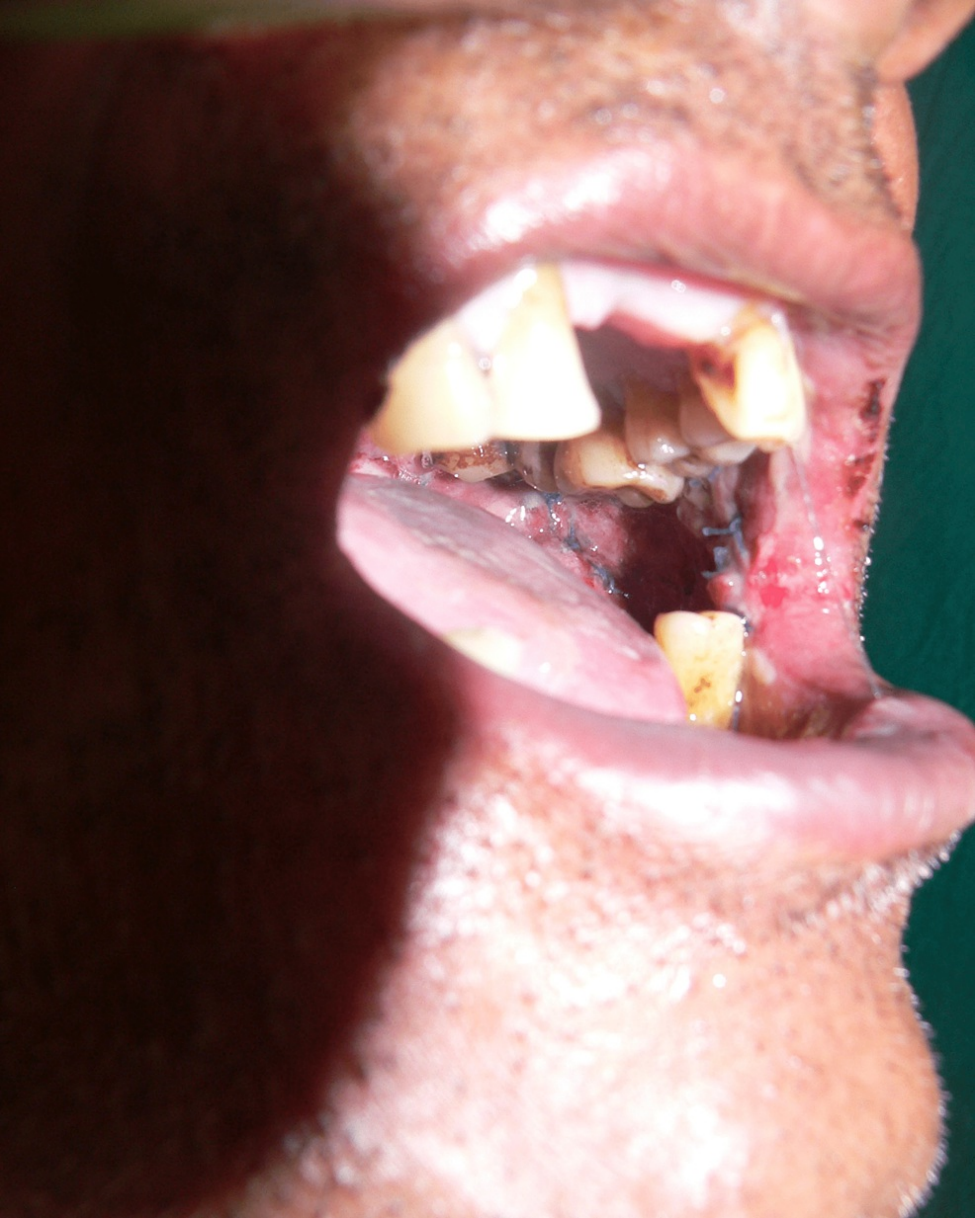
Picture of vertical fibrous bands, blanched, and erythematous mucosa before the treatment

**Figure 5:**
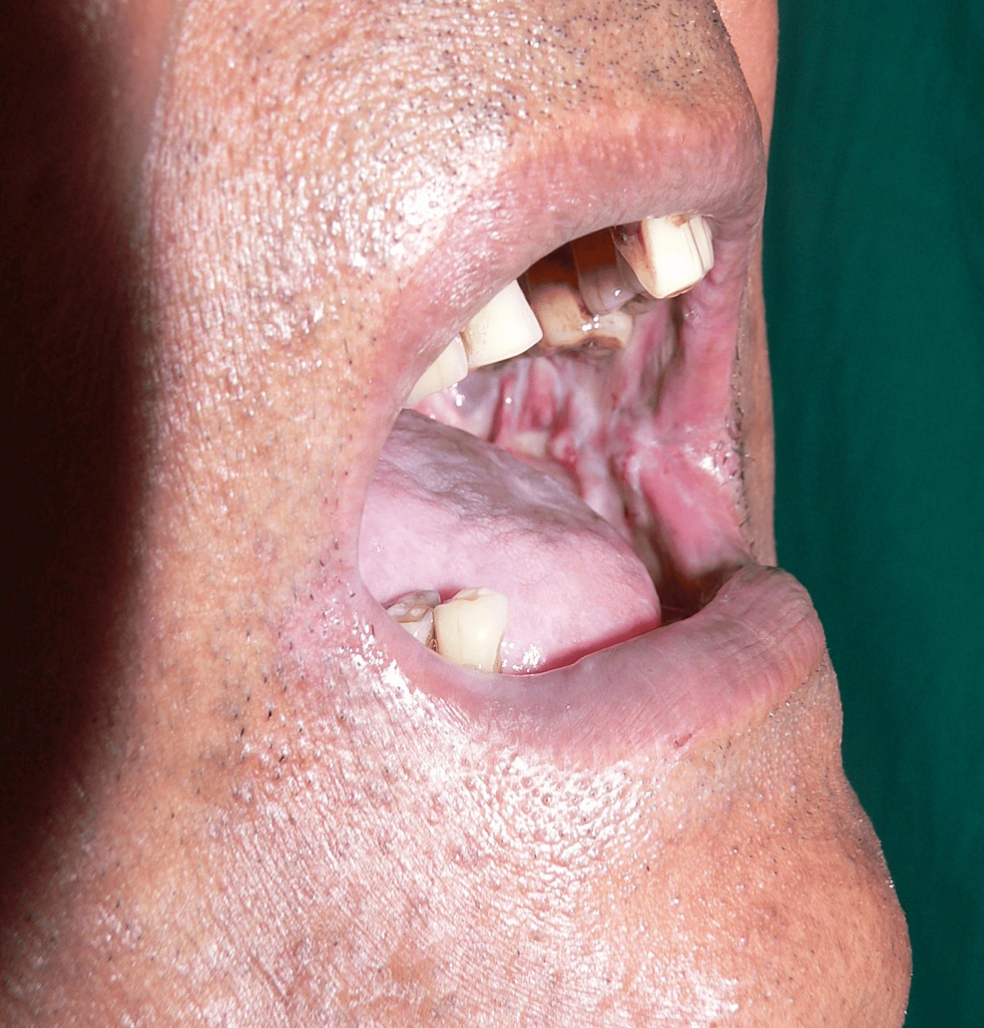
Post-treatment: 30-day follow-up picture of the fibrous bands, blanched, and erythematous mucosa

**Figure 6:**
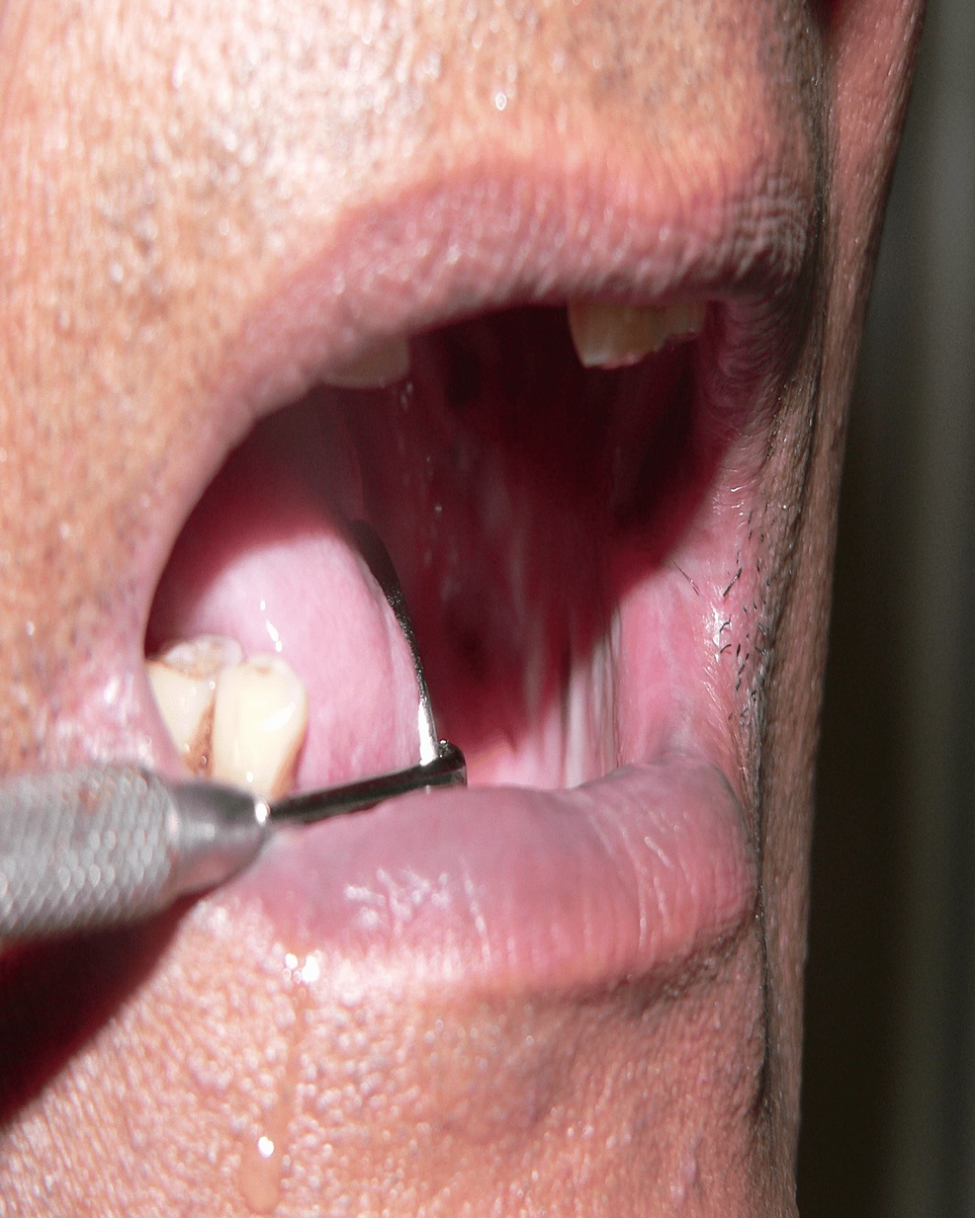
Post-treatment: 90-day follow-up picture showing marked reduction of the fibrous bands, blanched, and erythematous mucosa.

On the first visit, blanching was found in all 40 samples (100%) of all the study subjects and control subjects. In the last visit, blanching was found in 34 samples (85%) and was reduced in six subjects (15%). Blanching was present in all 40 subjects (100%) and was reduced in 0 subjects (0%) in the control subjects. There was a significant difference in blanching between the first visit and the last visit in the study subjects, with a statistically significant p-value of 0.0277* (Figures 4-6).

Table 9 compares the study subjects and control subjects regarding the status of the burning sensation at the first visit and the last visit.

**Table 9:** Comparison of study and control subjects regarding the status of burning at the first visit and the last visit Between the present and reduced in the last visit: Chi-square with Yate’s correction = 4.5051, p = 0.0341; Between the first visit and the last visit in the study subjects: Z = 5.0862, p = 0.000*; Between the first visit and the last visit in the control subjects: Z = 0.000, p = 1.0000

Visit	Burning	Study group	%	Control group	%	Total	%
First visit	Present	40	100.00	40	100.00	80	100.00
	Reduced	0	0.00	0	0.00	0	0.00
Last visit	Present	6	15.00	39	97.50	45	56.25
	Reduced	34	85.00	1	2.50	35	43.75
	Total	40	100.00	40	100.00	80	100.00

On the first visit, a burning sensation was present in all 40 subjects (100%), both study subjects and control subjects. On the last visit, a burning sensation was found in six subjects (15%) and reduced in 34 subjects (85%) of the study subjects. The burning sensation was found to be present in 39 subjects (97.50%) and reduced in one subject (2.50%) in the control subjects. The statistical analysis showed the difference regarding the status of the burning sensation in the first and last visits to be highly significant in study subjects with a p-value of 0.000 and not significant in control subjects with a p-value of 1.000.

Table 10 compares the study and control subjects with the status of vertical fibrous bands at the first and last visits. In the first visit, vertical fibrous bands were palpable in all 40 (100%) study subjects and control subjects.

**Table 10:** Comparison between the study subjects and control subjects regarding the status of vertical fibrous bands on the first and last visits Between present and reduced in the last visit: Chi-square with Yate’s correction = 1.3851, p = 0.232; Between the first and last visits in the study subjects: Z = 3.1797, p = 0.0014; Between the first visit and the last visit in the control subjects: Z = 0.0001, p = 0.9998

Visit	Vertical fibrous bands	Study subjects	%	Control subjects	%	Total	%
First visit	Normal	10	25.00	0	0.00	10	12.50
	Present	30	75.00	40	100.00	70	87.50
Last visit	Present	37	92.50	40	100.00	77	96.25
	Reduced	3	7.50	0	0.00	3	3.75
	Total	40	100.00	40	100.00	80	100.00

In the last visit, vertical fibrous bands were palpable in 37 (92.50%) and reduced in three (7.50%) study subjects. Vertical fibrous bands were palpable in all 40 (100%) control subjects. The statistical analysis was significant (p = 0.0014) in the study subjects and not significant in the control subjects (p = 0.99998) (Figures 4-6).

Table 11 compares the study and control subjects regarding the status of the uvula at the first visit and the last visit.

**Table 11:** Comparison of the study subjects and control subjects regarding the status of the uvula at the first visit and the last visit Between present and reduced in the last visit: Chi-squared = 0.7034, p = 0.4024; Between the first visit and the last visit in the study subjects: Z = 0.0001, p = 0.9998; Between the first visit and last visit in the control subjects: Z = 0.0001, p = 0.9998

Visit	Uvula	Study subjects	%	Control subjects	%	Total	%
First visit	Bud-shaped	9	22.50	7	17.50	16	20.00
	Normal	31	77.50	33	82.50	64	80.00
Last visit	Bud-shaped	10	25.00	6	15.00	16	20.00
	Normal	30	75.00	34	85.00	64	80.00
	Total	40	100.00	40	100.00	80	100.00

On the first visit, 31 (77.50%) had normal uvulas and nine (22.50%) had bud-shaped uvulas in the study subjects. In control subjects, 33 (82.50%) showed normal uvulas, and seven (17.50%) showed bud-shaped uvulas. In the last visit, nothing statistically significant was found in both the study and control subjects.

## Discussion

In this study, the majority of the patients were between 18 and 22 years of age, comprising about 30% of the study subjects and 35% of the control subjects, the mean age being 29.15+/−9.07 years and 27.03+/−7.59 years, respectively. The total mean age of the study sample was 28.09+/−8.38 years. This is per Kumar et al. [5], Hazarey V. K. et al. [6], and Sinor P. N. et al. [7], who reported 28 years, 28.8 years, and 29.1 years, respectively. The increase in the incidence of OSMF among the younger age group suggests a possible cause or reason for a change in the lifestyle of youngsters with the advent of attractive and conveniently packed sachets, mass/media advertisements, and the easy availability of gutka/pan masala. The present study showed a positive history of areca nut chewing in raw forms such as quid (13.75%) or commercial preparations such as gutka or pan masala (86.25%).

The most prevalent habit was commercially prepared areca nuts, which aligns with the findings of previous studies conducted by Kumar et al. [5], Sinor P. N. [7], and Sami A. M. et al. [8].

In this study, the mouth opening of the patients was measured by the inter-incisal distance between the incisal edges, which means the authors measured the distance between the edges of the upper and lower front teeth with the mouth fully opened. They compared the mouth-opening scores of the study subjects and the control subjects at the first and last visits of the study.

The results showed a statistically highly significant difference (p < 0.000*), which means that there was a significant change in mouth opening throughout the study for both groups.

After treatment, the researchers observed a significant improvement in mouth opening in the study subjects. On average, the mouth opening in the study subjects increased by 2.40 mm. The findings of this study regarding the improvement in mouth opening after treatment align with the results of previous studies conducted by Das et al. [9] and Hastak et al. [10], which also reported statistically significant improvements in mouth opening following treatment.

This study compared the tongue-protrusion scores between the study subjects and the control subjects at the first and last visits of the study. The statistical analysis showed that the difference in tongue-protrusion scores between the two groups was highly significant, with a p-value of 0.000*. This indicates that there were significant changes in tongue protrusion throughout the study. The study's findings regarding tongue-protrusion changes in the study and control subjects are per a previous study conducted by Shah et al. [11].

The results demonstrate a significant improvement in tongue protrusion in the study subjects between the first and last visits, with an average increase of 1.10 mm. On the other hand, there was no significant change in tongue protrusion among the control subjects between the two visits, as the mean difference was close to zero.

In this study, blanching was present in all 40 (100%) control subjects and study subjects on the first and last visits. Blanching was present in 34 (85%) and was reduced in six (15%) study subjects, whereas blanching was present in all 40 (100%) control subjects. The statistical analysis concluded that blanching significantly decreased from the first visit to the last visit for study subjects, per a study conducted by Aich et al. [12].

In this study, vertical fibrous bands were palpable in all 80 subjects. After treatment, that is, in the last visit, it was palpable in 37 (92.50%) and reduced in three (7.50%) study subjects, and was palpable in all 40 (100%) control subjects. Out of the total 80 subjects, 77 (96.25%) showed palpable vertical fibrous bands, and three (3.75%) showed reduction. Statistically, the analysis concluded that there was a reduction in the palpable fibrous bands post-treatment (Z = 3.1797, Z = 0,0014*) per a study conducted by Patel K. R. et al. [13].

In this study, all the subjects from both groups had a burning sensation. After treatment with the herbal paste formulation, a burning sensation was present in six (15%) and reduced in 34% of study subjects. It was present in 39 (97.50%) subjects and reduced in one (2.50%) among the control subjects. The statistical analysis of the data showed that the difference in the status of the burning sensation between pre-treatment and post-treatment was highly significant, with a p-value of 0.0000*.

In this study, the herbal paste formulation significantly reduced the burning sensation in the study subjects when compared to their pre-treatment status. Additionally, the presence of the burning sensation remained high in the control group, with minimal reduction observed in only one subject. The study states that the results are consistent with the findings of a study conducted by Srivastava et al. [14].

No clinically significant adverse reactions to the treatment were seen in the patients except that five patients experienced bad taste, a mild headache, and nausea, which disappeared after treatment.

Turmeric, tulsi, and honey have been used for centuries in traditional systems of medicine, such as Ayurveda and Unani, for their potential health benefits. These natural products have gained attention for their medicinal properties and therapeutic uses.

Turmeric is known for its active compounds called curcuminoids, with curcumin being the most studied. Curcumin, the main bioactive compound in turmeric, acts as a potent antioxidant and can neutralize or scavenge various reactive oxygen species produced by macrophages, helping to protect cells from oxidative damage [15]. Curcumin has anti-inflammatory properties and can inhibit cellular processes involved in inflammation, cellular proliferation, angiogenesis, and metastasis. It can also down-regulate certain proteins involved in apoptosis (programmed cell death) and metastasis. Due to these properties, turmeric is considered beneficial for conditions like inflammation, cancer, and various other health issues.

Tulsi, also known as holy basil, contains a diverse array of chemical constituents such as oleanolic acid, ursolic acid, rosmarinic acid, eugenol, carvacrol, and others. It is known for its antioxidant properties and ability to act as an adaptogen, relieving stress and enhancing immunity. Tulsi is believed to support a healthy metabolism and is considered a natural immunomodulator [16]. Its restorative properties make it a popular herbal remedy in traditional medicine.

Honey is considered an important medicine in the Ayurveda and Unani systems. According to Ayurveda, honey is classified into eight different types, each possessing unique properties and therapeutic benefits. The classification is based on the type of flower or plant from which bees collect nectar. The uses of honey in traditional medicine go beyond just topical applications. It is also used internally for treating conditions related to the oral cavity, such as gum problems and throat infections. It is known for its antibacterial properties and is often used to promote wound healing and provide relief from coughs and sore throats. Honey is also a source of antioxidants and is believed to have various health benefits.

Overall, traditional systems of medicine have utilized the above formulation for its potential therapeutic effects in managing conditions like inflammation, oxidative stress, immune modulation, and other health-related issues.

The results demonstrate the aspects discussed here. In demographics, the study primarily included subjects in the age group of 18-22 years, with the mean age being 28.09+/−8.38 years. There was a male predilection, with 96.25% of the participants being male. The habit of chewing gutka was the most predominant among all subjects. This suggests that gutka consumption was common among the participants.

The comparison of study subjects and control subjects showed statistically significant changes in various parameters in the study subjects compared to the control subjects in various areas. In mouth opening, there was a significant change in the study subjects compared to the control subjects, indicating that the intervention and treatment used in the study subjects affected the improvement or reduction in mouth opening. In tongue protrusion, significant changes were observed in the study subjects compared to the control subjects. This suggests that the intervention may have influenced tongue movement or position.

In terms of burning sensation, the study subjects experienced significant improvement compared to the control subjects. This indicates that the treatment affected the reduction or alleviation of the burning sensation reported by the participants. In blanching, there were significant changes between the study subjects and the control subjects. The treatment may have affected the blanching response in the oral mucosa. Regarding the reduction in palpable vertical fibrous bands, the study subjects demonstrated a significant reduction compared to the control subjects, suggesting that the treatment contributed to the reduction of fibrous bands in the oral cavity. Regarding the shape of the uvula, the intervention or treatment used did not have a noticeable impact on the appearance or shape of the uvula in either the study group or the control group.

The limitation of the study was the small sample size and significant gender imbalance, as there were only three female study subjects and no females were present in the control group. This could be present because women are much less likely than men to use tobacco in any form due to widespread social disapproval of women using tobacco. Sex role norms and general expectations concerning gender-appropriate behavior have also contributed to this discrepancy. A long-term follow-up study of the patients is required to fix the dose and duration of therapy to validate the use of such herbal medication in the treatment of OSMF and prevent malignant transformation. The methodological and ethical concerns raised indicate that more investigation with large sample size and a proper clinical trial setup is needed before conclusions are drawn about this herbal intervention for oral submucous fibrosis.

## Conclusions

Oral submucous fibrosis is a condition that affects the oral cavity and is characterized by the development of fibrous bands in the submucosal layers, leading to progressive difficulty in mouth opening and other symptoms. Numerous treatment modalities have been attempted for OSMF, ranging from vitamin supplements to various forms of steroids. However, due to the complexity of the disease, finding an effective and satisfactory treatment remains a challenge. This study focused on a novel herbal paste formulation containing turmeric, tulsi, and honey. The formulation showed promising results in managing oral submucous fibrosis and improving the signs and symptoms of patients. This herbal paste offers a non-invasive treatment, which can be beneficial for patients seeking alternatives to more aggressive interventions. The herbal ingredients used in the formulation have a long history of use in traditional medicine for their potential antioxidant, anti-inflammatory, analgesic, and immunomodulatory properties. This might contribute to their therapeutic effects in oral submucous fibrosis. While traditional medicine can offer valuable insights and potential treatments, rigorous scientific research and clinical studies are essential to establish the efficacy, safety, and optimal use of these natural products for specific medical conditions.
